# Correspondence

**Published:** 1875-06

**Authors:** A. A. Blount, Geo. Watt


Dr. J. Taft, Editor Dental Register.

*Dear Sir*:—My attention has been directed to a medicinal agent, which, after a very brief trial, gives results so satisfactory that I feel like communicating the suggestion to you, as it came to me, except that I will not furnish you the medicine ready for use, as my friend Dr. Blount did for me. The following letter speaks for itself:

"*Dear Sir*:—I have intended to write to you for some time, in regard to a new remedy that I have been using with much success and satisfaction, a substitute for carbolic acid, viz: "Salicylic acid." In my opinion (derived from a few weeks' experience) it is a much better antiseptic than carbolic acid or creosote, and is far from the objections attached to these medicines, I have been more successful in treating alveolar abscess, and other diseases of the mouth, than with any other remedy.

When used in simple solution take one part of the acid to three hundred parts of water. If a stronger solution is desired, take one part of the acid and three parts of phosphate of sodium to fifty parts of tepid water. Its advantages over all antiseptics are, that it is much more effective in smaller quantities, and in all quantities necessary for complete effectiveness entirely devoid of irritant action upon the living tissues. It is neither caustic nor corrosive in any quantity, and never produces inflammation, and has no poisonous effect in any reasonable quantity. It is said to prevent the processes of decomposition, which are beyond the reach of all other antiseptics.

I have only so far used the simple solution, one part acid to three hundred parts water which has been so effective that I had no desire to try the stronger solution.

In my office it has taken the place of phenol sodique for the reason that it accomplishes the same end, and is so much pleasanter, being free from taste or smell. I send you a little of the medicine, the simple solution, to try. I would send you more but have so little, this will do however to try, and by the time it is used up we can get a fresh supply. Write me soon.

Respectfully,

A. A. BLOUNT."

P. S. I do not know but it would be better to use the stronger solution in treating abscess of long standing."

Hoping you will help to investigate the subject,

I am yours,

GEO. WATT.

